# Structure–Property Relationships in Urea–Formaldehyde Bonded Napier Grass Composites: Influence of Lignocellulosic Chemistry on Interfacial Interaction and Thermal Stability

**DOI:** 10.1021/acsomega.6c03868

**Published:** 2026-08-10

**Authors:** Rahul Dev Bairwan, Goh Yan Ying, Che Ku Abdullah, Noorfarisya Izma Jeffri, Mohamad Shamil Faris Mohamad Khalid, Syeed SaifulAzry Osman Al Edrus, Nurul Fazita Mohammad Rawi

**Affiliations:** † Bioresource Technology Division, School of Industrial Technology, 26689 Universiti Sains Malaysia, Minden, Gelugor 11800, Malaysia; ‡ Green Biopolymer, Coatings and Packaging Cluster, School of Industrial Technology, Universiti Sains Malaysia, Gelugor, Penang 11800, Malaysia; § Adeante Agro Sdn. Bhd, Petaling Jaya, Selangor 47820, Malaysia; ∥ Faculty of Agrotechnology and Applied Science, i-CATS University College, Kuching 93350, Malaysia; ⊥ Institute of Ecosystem Science Borneo, Universiti Putra Malaysia Sarawak, Sarawak 97008, Malaysia

## Abstract

The valorization of lignocellulosic biomass into value-added materials requires a fundamental understanding of structure–property relationships and interfacial interactions within composite systems. In this study, Napier grass (*Pennisetum purpureum*) was investigated as a renewable biomass feedstock for the fabrication of urea–formaldehyde (UF) bonded composites. Particleboards were prepared at varying resin loadings (11%, 13%, and 15%), and their physical, mechanical, and thermal behavior was systematically evaluated. The results reveal that increasing UF content enhances modulus of rupture (MOR), internal bonding strength (IB), and thermal stability, while reducing water absorption and thickness swelling, indicating improved interfacial adhesion and reduced porosity. Fourier transform infrared spectroscopy (FTIR) analysis indicated enhanced resin coverage and interfacial interactions between the lignocellulosic particles and UF resin, which contributed to improved composite performance. However, excessive resin loading (15%) resulted in a reduction in modulus of elasticity (MOE) and impact strength relative to the 13% UF formulation, attributed to localized brittleness and overpenetration of resin into the fiber structure. The optimized formulation demonstrated comparable performance to wood-based particleboards, highlighting the influence of lignocellulosic composition on bonding efficiency and stress transfer. Overall, this work establishes a clear correlation between chemical composition, interfacial interactions, and macroscopic properties, demonstrating the potential of Napier grass as a sustainable feedstock for advanced biobased composite materials.

## Graphical abstract

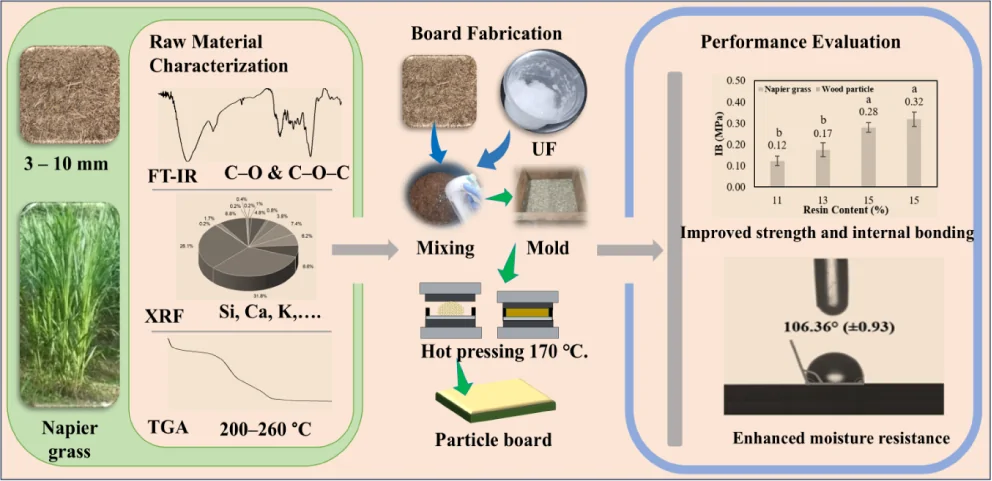

## 1 Introduction

The global demand for sustainable and cost-effective raw materials has accelerated the search for nonwood lignocellulosic alternatives in the wood-based panel industry. Particleboard, an engineered composite manufactured from wood or other fibrous materials bonded with synthetic resin under heat and pressure is widely used in interior construction, furniture, and decorative applications. With rising population and rapid industrialization, the availability of solid wood has declined, while its cost has steadily increased, intensifying the need for renewable feedstocks for composite fabrication.^1^ According to Inkwood Research (2024), Malaysia’s wood-based panel market is projected to grow at a CAGR of 6.98% between 2024 and 2032, reaching USD 8.24 billion by 2032, driven by increasing residential and commercial furniture demand.^2^ However, this growth trend also underscores the urgency of developing sustainable raw materials that reduce reliance on forest-derived timber.

Nonwood fibers and agricultural residues have emerged as promising renewable alternatives due to their abundance, low cost, and high cellulose content. Numerous studies have investigated agro-based materials such as kenaf^3^, bamboo^4^, corn husk and stalks^5,6^ , hemp^1^, and sugar cane bagasse^7^ for composite board manufacturing. These materials often possess mechanical and physical properties comparable to wood while offering environmental advantages such as reduced carbon footprint and waste valorization potential. Among them, Napier grass (*Pennisetum purpureum*), also known as elephant grass has drawn significant attention as a fast-growing perennial grass species suitable for tropical climates, such as Malaysia’s.

Napier grass has long been cultivated as animal fodder but is now increasingly explored for industrial and environmental applications due to its rapid growth rate (up to 4–7 m in height), high lignocellulosic content, and adaptability to diverse soil conditions.^8,9^ The grass possesses a well-developed root system that aids in soil conservation and erosion control^10^, while its high cellulose-to-lignin ratio makes it an attractive candidate for pulp, paper, and composite production.^11^ Recent works also highlight its potential as reinforcement in biodegradable and hybrid composites for packaging and automotive applications due to its good mechanical strength and antibacterial performance.^12,13^ These attributes position Napier grass as a sustainable and locally available raw material for producing particleboards that can substitute wood.

The performance of particleboards, however, depends not only on the fiber type but also on the adhesive system used. Urea-formaldehyde (UF) resin remains the most widely used adhesive in the global wood panel industry due to its low cost, rapid curing, and high bonding strength.^14^ UF accounts for approximately 91% of adhesives used in wood-based composites such as plywood, MDF, and particleboards.^15^ Although UF resin remains the dominant adhesive in particleboard manufacturing due to its low cost and excellent bonding performance, concerns regarding formaldehyde emissions continue to drive the development of low-emission and biobased adhesive alternatives. To mitigate these challenges, recent research has explored UF modifications using nanocellulose, lignin, tannins, and other biobased additives to enhance water resistance and reduce formaldehyde release.^16,17^ Beyond macroscopic properties, composite performance is governed by interactions between lignocellulosic fibers and thermosetting resins. Napier grass contains abundant hydroxyl groups from cellulose and hemicellulose, which can interact with amide functionalities of UF resin during curing. These interactions, primarily through hydrogen bonding and physical adhesion, influence interfacial bonding, moisture resistance, and thermal stability, thereby establishing a structure–property relationship within the composite system.

Resin content is another crucial factor influencing the overall quality of particleboards. Increasing resin loading typically improves bonding, internal bond (IB) strength, modulus of rupture (MOR), and dimensional stability by reducing water absorption and thickness swelling.^5,18^ However, excessive resin usage may lead to brittleness, higher costs, and reduced elasticity. Researchers reported that an optimal resin content range enhances mechanical and thermal properties without compromising flexibility or sustainability.^7^ Therefore, optimizing resin content for new raw materials such as Napier grass is critical to achieving a balance between performance, cost, and environmental considerations. While several studies have explored nonwood materials for particleboard production, limited work has focused on Napier grass in structural board applications, particularly regarding resin content optimization. In addition, the relationship between lignocellulosic composition, interfacial interactions with UF resin, and resulting mechanical and thermal properties remains insufficiently understood.

In this study, Napier grass particles were used to fabricate urea–formaldehyde bonded composites at varying resin contents. The effects of resin loading on physical, mechanical, and thermal properties were systematically evaluated, with particular emphasis on correlating interfacial interactions and microstructural features with macroscopic performance. The optimized formulation was further compared with a wood-based particleboard fabricated under identical conditions to assess the influence of lignocellulosic composition on bonding efficiency and structural behavior.

## 2 Materials and Methods

### 2.1 Raw Materials

Napier grass (*Pennisetum purpureum*) was collected in June 2024 from a cultivation site in Kuala Lumpur, Malaysia, and supplied by Circafeed Sdn. Bhd. Commercial urea–formaldehyde (UF) adhesive was obtained from Asta Chemicals Sdn. Bhd., while mixed hardwood particles used for comparison were provided by the Forest Research Institute Malaysia (FRIM), Kuala Lumpur, (Malaysia). The UF resin had a solid content of 53.04%, a pH of 8.0, and exhibited a milky-white appearance.

### 2.2 Preparation of Raw Materials

Fresh Napier grass was first cleaned to remove dirt and impurities, cut into small pieces, and subsequently processed using a grinding machine supplied by a local fabricator (HH Saintifik Ent., Penang, Malaysia) to produce coarse particles. The particles were sieved to obtain a size range of 3–10 mm, suitable for particleboard fabrication. The sieved particles were oven-dried at 50 °C overnight to reduce the moisture content to below 10%. Hardwood particles were prepared following the same procedure to ensure consistency in particle size and moisture content before use.

### 2.3 Fabrication of Particleboards

Napier grass particleboards were fabricated using urea–formaldehyde (UF) resin contents of 11%, 13%, and 15% (w/w), based on the oven-dried weight of the Napier grass particles. The boards were designed with a target density of 750 kg m^–3^ and dimensions of 200 mm × 200 mm × 5 mm. Before blending, the viscous UF adhesive was diluted with 10 mL of hot water to improve flowability. The resin was then gradually added to the Napier grass particles and mixed using an electric hand mixer to ensure uniform resin distribution. The adhesive-coated particles were placed into a wooden forming box with a metal mold beneath to form the mat. Hot pressing was carried out using a hydraulic hot press at 170 °C. The mats were first prepressed twice for 5 min each, followed by a final hot pressing for 20 min. After pressing, the boards were cold-pressed for 5 min to relieve internal stresses and facilitate cooling. Two replicate Napier grass particleboards were produced for each resin content. or comparison, two wood-based particleboards were fabricated from mixed hardwood particles supplied by the Forest Research Institute Malaysia (FRIM), Kuala Lumpur, Malaysia, under identical manufacturing conditions using 15 wt % UF resin content. All fabricated boards were conditioned at room temperature (25 ± 2 °C) for 24 h prior to cutting and subsequent testing.

### 2.4 Characterization of Napier Grass Raw Material

#### 2.4.1 Particle Density and Specific Gravity

The particle density and specific gravity of the napier grass samples was measured using a Micromeritics AccuPyc 1340 gas pycnometer following the ISO 12154 standard. Helium was employed as the analysis gas. Prior to sample measurement, the AccuPyc 1340 was calibrated using a stainless-steel reference volume. The 10 cm^3^ sample cell was also calibrated and subsequently used for the analysis. Samples were loaded into the cell, and their masses were recorded. The measurement procedure involved five cycles of helium gas purging, followed by five measurement cycles to determine the sample volume. The density of each sample was then calculated using the measured volume and the known mass.

#### 2.4.2 Chemical Composition

Chemical composition analysis was conducted to characterize the biochemical constituents of Napier grass, according to TAPPI standard, T204 cm-97.^19^ The contents of extractives, holocellulose, hemicellulose, and lignin were quantified, as these components significantly influence the bonding efficiency, dimensional stability, and thermal behavior of particleboards. In each case, five samples were used and the average values were reported. Volatile matter on a dry basis was quantified following BS EN 15148, while ash content was measured according to BS EN 14775, used in a similar study.^20^

#### 2.4.3 Fourier Transform Infrared Spectroscopy (FTIR)

The functional groups and chemical bonding characteristics of Napier grass were analyzed using a (PerkinElmer, Model PC1600, PerkinElmer, Inc., Waltham, MA, USA). FTIR measurements were performed in transmittance mode using the KBr pellet method. Samples were scanned over the 400–4000 cm^–1^ spectral range with a resolution of 4 cm^–1^ and 32 accumulated scans. The acquired spectra were subjected to baseline correction and smoothing using the instrument software prior to analysis.

#### 2.4.4 X-ray Fluorescence (XRF)

The elemental composition of the Napier grass was determined using X-ray fluorescence (XRF) analysis in the Earth Material Characterization Laboratory, Universiti Sains Malaysia, Penang, Malaysia. A representative portion of each sample was first ground to approximately 50 μm using a motorized grinding machine and then manually milled to a finer particle size of 20 μm, suitable for X-ray fluorescence (XRF) analysis. For XRF specimen preparation, 0.5 g of the sample was mixed with 5.0 g of Spectroflux, ignited at 1100 °C for 20 min, and subsequently cast into a glass disc with a diameter of 32 mm. The specimens were analyzed for 10 major elements using a fully automated Panalytical Axios Max (Netherlands) XRF spectrometer under a standard elemental configuration. Calibration was performed using wide-range oxide certified standard reference materials to construct the curves for all 10 elements.

#### 2.4.5 Thermogravimetric Analysis (TGA)

Thermal stability and degradation behavior were examined using a Mettler Toledo thermogravimetric analyzer (Mettler Toledo, Schwarzenbach, Switzerland). Approximately 10 mg of Napier grass particles were placed in an alumina pan and heated from 30 to 800 °C under a nitrogen atmosphere at a constant heating rate of 10 °C/min. The mass loss profile and corresponding thermal events were recorded to evaluate the thermal characteristics of the raw material.

### 2.5 Characterization of Particleboards

#### 2.5.1 Physical Characterization

The physical properties of the particleboards density, water absorption (WA), and thickness swelling (TS) were determined in accordance with the Japanese Industrial Standard JIS A 5908:2003. For density measurement, three specimens were cut from each board, and their mass was measured using an analytical balance. Dimensions were determined with a digital calliper to obtain an average reading from three measurements. The density (ρ) of the particleboard specimens was calculated using eq 1:

1 $$Density=\frac{mass\;\left(kg\right)}{volume\;\left(m^{3}\right)}$$

where *m* is the mass (kg) and *V* is the volume (m^3^).

To evaluate WA and TS, three specimens (50 mm × 50 mm) from each resin formulation were dried, weighed, and measured prior to immersion in distilled water at room temperature (25 °C) for 24 h. After immersion, excess surface water was wiped off before recording the final mass and thickness. The WA and TS were calculated using eqs 2 and 3, respectively:

2 $$Water\;absorption\;\left(\%\right)=\frac{final\;weight\;\left(g\right)-initial\;weight\;\left(g\right)}{initial\;weight\;\left(g\right)}\times 100$$

3 $$Thicknesss\;welling\;\left(\%\right)=\frac{final\;thickness\;\left(mm\right)-initial\;thickness\;\left(mm\right)}{initialthickness\;\left(mm\right)}\times 100$$

All physical and mechanical property measurements were performed using three replicates (n = 3), and the results are reported as mean ± standard deviation.

The water contact angle (WCA) of the particleboards was measured at room temperature using an Attension Theta Lite Optical Tensiometer (Biolin Scientific, Sweden). A 2 μL water droplet was deposited on the surface with an automatic microsyringe, and each sample was measured three times, with the results averaged.

#### 2.5.2 FTIR Analysis

FTIR analysis of the Napier/UF particleboards was performed following the procedure described in Section 2.4.3.

### 2.6 Mechanical Testing

Mechanical performance was assessed according to JIS A 5908:2003, including modulus of rupture (MOR), modulus of elasticity (MOE), internal bond (IB) strength, and impact resistance.

For bending tests, specimens of 200 mm × 50 mm were tested using an Instron Universal Testing Machine under a three-point bending configuration with a span length of 150 mm and crosshead speed of 1 mm min^–1^. MOR and MOE were determined from the load–deflection data, and the mean values from three replicates were reported. For IB testing, square specimens of 50 mm × 50 mm were bonded between two metal loading blocks using adhesive and fully cured before testing. The samples were loaded in tension using the Instron machine at a crosshead speed of 2 mm min^–1^ until failure, and the average IB strength was recorded from three replicates. The impact strength was evaluated using the Izod notched test following ASTM D256, employing a GOTECH GT-7045-MDL impact tester. Specimens measuring 63.5 × 12.5 mm were machined with a V-notch of 90° and a depth of 2 mm prior to testing. Impact strength was measured on specimens of 67.5 mm × 10 mm using a pendulum impact tester with a 1 J hammer energy. The absorbed energy during fracture was recorded, and impact strength was calculated as the average of three samples for each resin content. Statistical analysis was performed for the mechanical properties (MOE, MOR, IB, and impact strength) using SigmaPlot 12.0. All quantitative measurements were performed in triplicate (n = 3), and the results are presented as mean ± standard deviation. One-way analysis of variance (ANOVA) followed by Tukey’s HSD post hoc test was used to determine significant differences among formulations at a significance level of *p* < 0.05, where applicable.

### 2.7 Digital Microscopy

Fracture surfaces were observed under a digital microscope using Keyence VHX Digital Microscope, in the Earth Material Characterization Laboratory, Universiti Sains Malaysia, Penang, Malaysia, to study particle alignment and particle pull-out behavior.

### 2.8 Thermal Characterization

Thermal stability of the particleboards was analyzed using thermogravimetric analysis (TGA). Approximately 10 mg of powdered sample was placed in an alumina crucible and heated from 30 to 800 °C at a heating rate of 10 °C min^–1^ under a nitrogen atmosphere. The onset degradation temperature (T_onset_) and residual weight (%) were determined to assess the thermal behavior of the boards at different resin contents.

## 3 Results and Discussion

### 3.1 Raw Material Properties

The physical properties of 3 mm Napier grass particles were characterized in terms of density and specific gravity to evaluate their suitability as a reinforcing filler. As shown in Figure 1a, the average density of the particles was 0.894 g/cm,^3^ while the specific gravity was 0.897, indicating closely matched values and low variability. Both measurements were obtained from five repeated cycles of sample volume determination, ensuring reproducibility. The figure presents the density and specific gravity side by side as bar graphs with standard deviation error bars. The minimal difference between density and specific gravity suggests that the particles are compact with low porosity and negligible trapped air or moisture effects. Such uniformity in physical properties is advantageous for composite processing, as it promotes consistent packing, interfacial adhesion, and mechanical performance. The measured values are consistent with typical lignocellulosic biomasses, confirming that Napier grass is a promising candidate for biocomposite reinforcement.^21,22^

**Figure 1:**
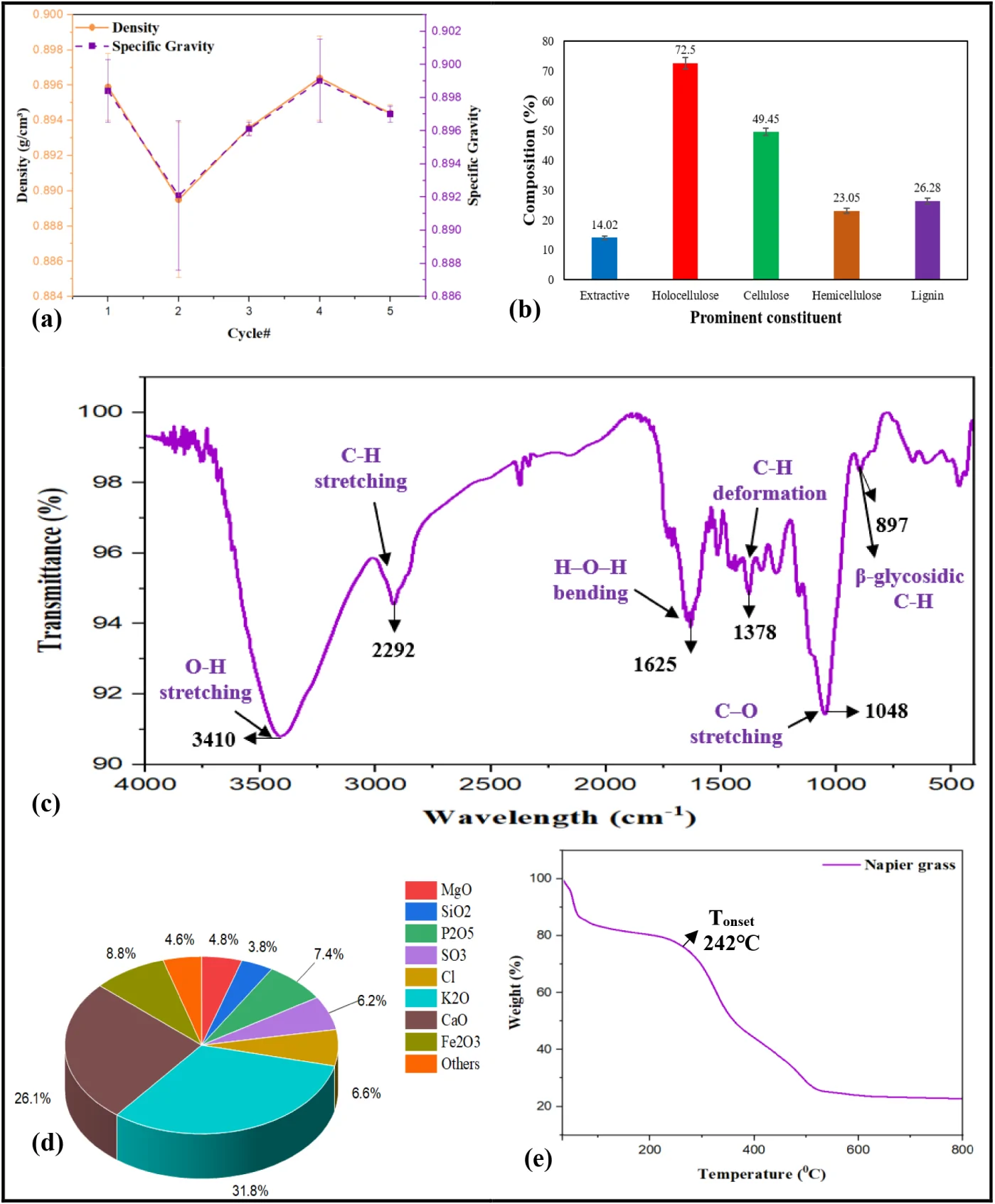
(a) Density and specific gravity of Napier grass particles, (b) Chemical composition showing extractives, holocellulose, hemicellulose, and lignin contents, (c) FTIR transmittance spectrum (400–4000 cm^–1^), (d) XRF elemental composition of Napier grass, and (e) Thermogravimetric analysis (TGA) curve.

The chemical composition of Napier grass (Figure 1b) reveals a lignocellulosic structure well-suited for engineered biobased composites. The holocellulose content was high at 72.50%, indicating that Napier grass is rich in structural carbohydrates. Within this fraction, cellulose accounted for 49.45%, a value comparable to or higher than many tropical grasses and agricultural residues commonly used in composite fabrication. High cellulose content contributes positively to mechanical reinforcement, stiffness, and fiber integrity during board pressing.^23^ The hemicellulose content of 23.05% falls within the typical range for fast-growing tropical grasses and contributes to the flexibility and amorphous character of the fiber structure. While hemicellulose is more hydrophilic and thermally unstable than cellulose, its proportion here remains suitable for composite manufacturing. The lignin content of 26.28% is substantial relative to other grassy biomass and lies within the reported range for *Pennisetum purpureum*. Elevated lignin contributes to thermal resistance, higher char formation, and improved rigidity during hot pressing.^24^ Lignin’s hydrophobic nature can also moderately reduce water uptake, benefiting dimensional stability of the resulting particleboards. The extractive content of 14.02% reflects the presence of waxes, fatty acids, phenolics, and soluble organics commonly found in fast-growing grasses. Extractives can influence resin penetration and interfacial bonding by modifying fiber surface energy. Several studies note that extractive-rich fibers may require higher adhesive loading to achieve optimal bonding due to surface inactivation or hydrophobic barriers. Taken together, the chemical composition of Napier grass balanced in cellulose, moderate in hemicellulose, rich in lignin, and with manageable extractives supports its suitability as a sustainable raw material for UF-bonded particleboards. The combination of mechanical reinforcement potential and lignin-derived thermal stability makes Napier grass an attractive biomass resource for biobased panel manufacturing, especially within the biocircular economy.

The FTIR spectrum of Napier grass (Figure 1c) displays characteristic vibrational bands of lignocellulosic biomass that confirm a cellulose-rich material with measurable hemicellulose and lignin fractions. A broad, strong band at 3410 cm^–1^ corresponds to O–H stretching from hydroxyl groups in cellulose and hemicellulose, indicating a hydrophilic surface prone to moisture uptake and hydrogen-bonding interactions with adhesives. This feature is commonly reported for grass and straw feedstocks and is directly related to water-absorption behavior in boards.^25^ The C–H stretching vibrations at 2922 cm^–1^ further corroborate the presence of aliphatic polysaccharide chains. In the fingerprint region, the band near 897 cm^–1^ is assigned to β-glycosidic linkages of cellulose (an anomeric C–H vibration), which is a diagnostic band for cellulose crystallinity and polymer backbone integrity. The intense band at 1048 cm^–1^ is attributed to C–O and C–O–C stretching of cellulose and hemicellulose, signaling abundant accessible hydroxyl-bearing polysaccharide sites that can interact with UF resin via physical adsorption or hydrogen bonding.^26^

Bands at 1260 and 1378 cm^–1^ are consistent with C–O stretching and CH deformation modes often associated with hemicellulose and lignin side groups, while the aromatic skeletal vibration around 1513–1516 cm^–1^ indicates the presence of lignin (aromatic ring vibrations). The detection of these lignin-related bands explains, in part, the relatively good thermal stability of the material and its contribution to panel rigidity.^27,28^ Minor features at low wavenumbers (≈465–666 cm^–1^) likely reflect skeletal vibrations and may indicate inorganic residues (e.g., silicate contaminants) that are frequently observed in grasses and that can be confirmed by complementary XRF data. A weak band near 2372 cm^–1^ is most likely an instrumental CO_2_ artifact and does not represent biomass chemistry. The FTIR profile confirms the expected lignocellulosic composition of Napier grass (cellulose-rich with hemicellulose and lignin), and helps explain the material’s behavior in hot-pressed particleboards: abundant hydroxyl and C–O sites favor resin wetting and bond formation, whereas the lignin fraction contributes to thermal resistance and stiffness factors that together underpin the trends observed in resin-dependent mechanical and dimensional properties.

The XRF analysis of Napier grass (Figure 1d) revealed the presence of several inorganic constituents commonly observed in grass-derived biomass, with major signals attributable to Si, Ca, K, Al, Fe, Mg and P (XRF report). Such an elemental fingerprint is consistent with previous characterizations of elephant/Napier grass ashes and other herbaceous feedstocks, which typically show silica as a dominant oxide together with appreciable alkali (K) and alkaline-earth (Ca, Mg) elements.^29,30^ The occurrence of silica (SiO_2_) is particularly important: while silica can act as an inert filler that may improve stiffness and dimensional stability of panels, elevated silica levels are also associated with increased abrasiveness during mechanical processing (tool wear) and may influence resin penetration and surface finish.^31^

Alkali and alkaline-earth metals such as potassium and calcium, identified by XRF, have a strong influence on thermal behavior because they lower ash melting temperatures and can form low-melting eutectic salts (e.g., potassium/calcium silicates) under heating. Such transformations can modify hot-pressing behavior (e.g., promote localized softening or eutectic flow) and affect board integrity if not controlled.^32^ In practice, the moderate inorganic load observed here complements the low total ash content measured by proximate analysis (2.26%), indicating that although inorganic species are detectable, they are not present in quantities likely to dominate bulk board properties. Nonetheless, even trace mineral species can affect adhesive bonding and thermal decomposition pathways (for example, by catalyzing char formation or altering resin cure chemistry), and therefore should be considered when optimizing pressing schedules and adhesive formulations.^33^ Taken together, the XRF results suggest that Napier grass offers a typical grass-type mineral profile that will not preclude its use in UF-bonded particleboards, but may require modest processing adjustments (particle cleaning, fine sieving, or pretreatments) or selection of adhesive systems tolerant to mineral-rich surfaces to minimize tool wear and ensure uniform bonding. Similar strategies have been recommended in the literature when incorporating agro-residues with elevated silica or alkali contents into composite boards.

The thermogravimetric profile of Napier grass (Figure 1e) exhibits the characteristic multistage degradation pattern typical of lignocellulosic biomass. An initial minor weight loss (∼2–4%) occurs below 120 °C, corresponding to evaporation of loosely bound moisture and volatile extractives. The first major decomposition stage begins around 220–280 °C, associated primarily with hemicellulose degradation due to its amorphous and thermally unstable structure. This is consistent with the reported thermal behavior of hemicellulose in tropical biomass, which typically decomposes between 210 and 300 °C.^34^ A more pronounced weight-loss event follows in the range of 300–380 °C, indicating the breakdown of cellulose, whose crystalline structure yields a sharper and more intense decomposition peak. Beyond 400 °C, the mass loss becomes gradual, representing the slow and broad decomposition of lignin, due to its heterogeneous aromatic structure.^35^

The final residual mass at 800 °C reflects the ash content and mineral constituents inherent in Napier grass, which tend to be higher in herbaceous biomass compared to wood. Elevated ash formation aligns with previous findings reporting substantial inorganic content in Napier grass, including silica, potassium, and calcium oxides (Li et al., 2023). Overall, the TGA profile indicates that Napier grass possesses thermal stability comparable to other high-cellulose energy crops, supporting its suitability as a raw material for composite boards and applications within the biomass sector.

### 3.2 Properties of Particleboards

This section presents the physical, mechanical, and thermal performance of Napier grass particleboards manufactured with 11%, 13%, and 15% UF resin content, followed by a comparative evaluation against wood particleboards fabricated at the optimum resin loading. The results provide insight into how resin content influences interfacial adhesion, moisture resistance, stiffness, strength, and thermal stability of Napier grass as an alternative feedstock for particleboard production.

### 3.3 Physical Properties

The density values of Napier grass particleboards produced with 11%, 13%, and 15% UF resin, along with wood particleboards fabricated at 15% resin content, are presented in Figure 2a. All boards showed only minor deviations from the targeted density of 750 kg/m^3^, with differences ranging from 2 to 19 kg/m^3^. This indicates that the fabrication process including mat formation, pressing pressure, and moisture control was well regulated. Maintaining density close to the target is essential, as density strongly governs the physical and mechanical behavior of particleboards. Higher density typically results in improved interparticle contact and reduced void volume, which enhances resin distribution and consolidation during hot pressing. As reported by Ramezanpoor Maraghi et al. (2018), increased board density leads to lower water absorption and improved mechanical performance such as MOE, MOR, and IB.^18^ More recent studies also confirm that density is a critical determinant of panel quality: Wimmer et al. (2023) demonstrated that uniform density enhances stress transfer efficiency within lignocellulosic composites, while optimum compaction reduces thickness swelling and improves dimensional stability.^36^ Given these established relationships, setting a constant targeted density of 750 kg/m^3^ ensures a fair and meaningful comparison between Napier grass particleboards and conventional wood particleboards. This approach isolates the influence of resin content and raw material characteristics without the confounding effects of density variation.

**Figure 2:**
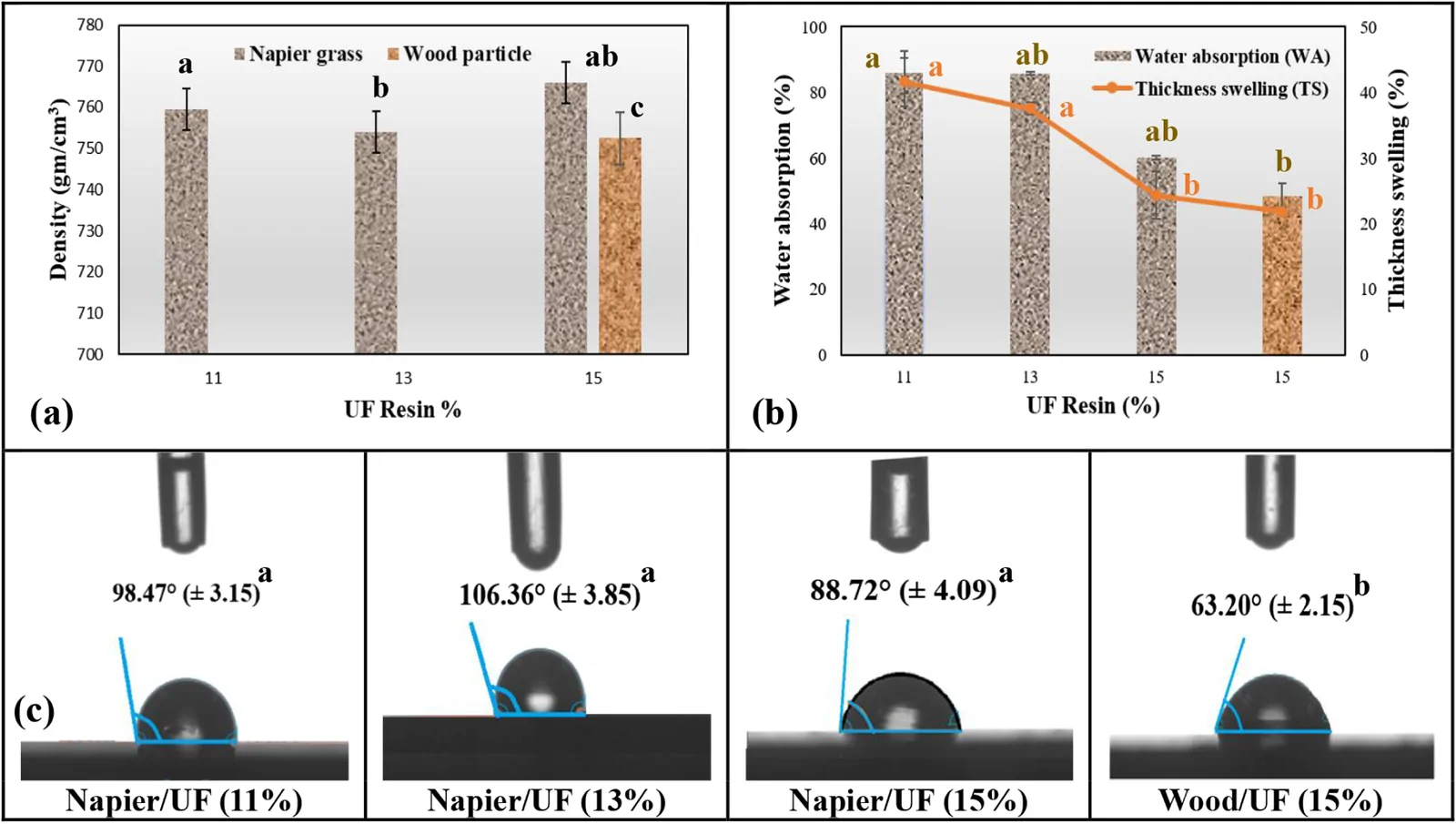
(a) Density of particleboards, (b) Water absorption and **t**hickness swelling after 24 h immersion, and (c) Water contact angle measurements for Napier grass and wood particle/UF particleboards.

The water absorption behavior of Napier grass particleboards manufactured with 11%, 13%, and 15% UF resin content, together with wood particleboards at 15% resin content, is shown in Figure 2b. A clear decreasing trend was observed as the resin content increased: water absorption reduced from 86.26% at 11% resin to 60.10% at 15% resin, reflecting improved particle encapsulation and reduced availability of hydrophilic sites at higher resin loadings. Similar observations have been reported in previous studies, where higher UF loading enhanced particle surface sealing and reduced moisture ingress.^3^ At equivalent resin content (15%), the wood particleboard showed lower water absorption (48.27%). The lower water absorption observed for the wood particleboard may be associated with differences in particle morphology and board structure. However, since detailed characterization of the wood raw material was beyond the scope of this study, the comparison is intended primarily as a performance benchmark under identical fabrication conditions. In contrast, Napier grass contains abundant hydroxyl-rich polysaccharides and features a more open, vascular microstructure, contributing to elevated moisture sorption.^37^ These intrinsic characteristics explain why Napier-based boards consistently exhibit higher water absorption despite improved resin coverage at higher UF levels. The results further confirm that increasing resin content improves dimensional stability by reducing accessible voids and enhancing interface consolidation between particles and UF resin. Recent findings by Pędzik et al. support that the reduction of porosity and improved adhesive distribution are key determinants in limiting water transport in lignocellulosic composites.^38^ Although PF adhesive systems offer superior water resistance compared to UF resins, the present results demonstrate that Napier grass can achieve acceptable water absorption values with sufficient UF resin loading, making it viable for nonstructural interior applications.

The thickness swelling (TS) values of Napier grass particleboards produced with 11%, 13%, and 15% UF resin content, alongside wood particleboards fabricated at 15% resin loading is depicted in Figure 2b. A clear decreasing trend was observed for Napier grass boards as resin content increased, indicating that higher UF levels improve dimensional stability. This behavior is typical in lignocellulosic composites, since greater resin coverage reduces void volume, enhances particle encapsulation, and limits pathways for moisture diffusion. Similar reductions in TS with higher UF content have been reported for agricultural residue-based particleboards, including corn stalk and bamboo composites.^4,6^ Thickness swelling in particleboards primarily arises from the hygroscopic nature of cellulose-based materials. Water molecules penetrate cell walls and form hydrogen bonds with hydroxyl groups in cellulose and hemicellulose, causing matrix expansion.^39^ When resin content is low, more hydrophilic sites remain exposed, and voids between particles facilitate water transport into the board, promoting pronounced swelling. Conversely, at higher resin levels, UF polymer fills a larger proportion of these voids and forms a more continuous network around the particles, restricting water ingress and limiting the extent of fiber swelling. The TS results therefore reinforce the importance of achieving adequate resin distribution and particle coverage during mat formation. Despite using the same resin content (15%), the wood particleboard exhibited lower thickness swelling than the Napier grass particleboard. This result indicates better dimensional stability under the conditions employed in this study. The difference may be related to variations in particle morphology, internal structure, and resin distribution between the two lignocellulosic materials. Although increasing UF resin content significantly improved the dimensional stability of Napier grass boards, further improvements may be achieved through fiber pretreatment, densification, or hybrid particle formulations.

Water contact angle (WCA) measurements (sessile-drop method, n = 3 per condition) revealed clear and material-dependent differences in surface wettability (Figure 2c). Napier grass particleboards exhibited a strong dependence of surface hydrophobicity on resin loading. The 13% UF boards displayed the highest mean contact angle (106.36° ± 3.85°), indicating a distinctly hydrophobic surface, whereas the 11% UF boards were, on average, hydrophilic (98.47° ± 3.15°) and the 15% UF boards were close to the hydrophobic/hydrophilic threshold (88.72° ± 4.09°). By contrast, the wood-based particleboard at 15% UF was clearly hydrophilic (63.20° ± 2.15°). The increase in WCA from 11% → 13% UF is consistent with improved resin coverage of particle surfaces and a relative reduction in accessible polar OH sites: additional resin fills surface voids and increases the proportion of nonpolar C–H groups at the interface, thereby reducing surface energy and increasing contact angle. This effect of resin loading on wettability has been observed previously for lignocellulosic scrimber and particleboard systems.^40,41^

The relatively low standard deviations observed for all formulations indicate good reproducibility of the contact angle measurements and reasonably uniform surface characteristics. Surface roughness and porosity nevertheless influence the apparent contact angle, as rougher or more porous surfaces may promote air entrapment or liquid penetration, thereby affecting droplet spreading behavior.^42,43^ Comparing Napier grass to wood particleboards, the higher WCA values observed for Napier grass boards, particularly at 11% and 13% UF resin contents, are consistent with their improved resistance to moisture uptake. Increased surface hydrophobicity reduces the accessibility of polar hydroxyl groups and limits capillary water penetration, resulting in lower water absorption and enhanced dimensional stability. This relationship between surface wettability and moisture resistance has been widely reported for lignocellulosic particleboard systems.^44,45^

### 3.4 FTIR Analysis

The FTIR spectra of Napier grass particleboards bonded with 11%, 13%, and 15% UF resin, together with a wood particleboard prepared at 15% resin content, are presented in Figure 3. All samples exhibit the characteristic absorption bands of lignocellulosic biomass and urea–formaldehyde adhesive, indicating that hot-pressing did not cause major chemical degradation of fibers. The broad −OH stretching band at 3300–3400 cm^–1^, associated with cellulose, hemicellulose, and lignin, is prominent across all formulations.^46^ A slight reduction in intensity with increasing UF content suggests partial masking of hydrophilic hydroxyl groups due to improved resin coverage on the particle surfaces and possible involvement of −OH groups in hydrogen bonding interactions with UF functional groups. The C–H stretching band at ∼2900 cm^–1^, representing CH and CH_2_ groups in carbohydrates and lignin, remained relatively unchanged, confirming that the bulk chemical structure of the fibers was preserved during processing.^47^ Distinctive UF-related bands were observed between 1650 and 1550 cm^–1^, corresponding to amide (N–H and C=O) vibrations from the cured UF network. These bands became more intense at higher resin loadings (13–15%), indicating a higher concentration of UF polymer deposited on the fiber surface and more complete curing associated with the formation of a cross-linked UF network through methylene and dimethylene ether linkages.^48^ A similar trend was noted in the wood particleboard, where stronger amide absorption reflected better fiber–resin compatibility, consistent with its superior mechanical performance. In the carbohydrate fingerprint region (1200–900 cm^–1^), characteristic C–O–C and C–O stretching bands showed marginal reductions in intensity with increasing UF content, again suggesting increased resin encapsulation and reduced exposure of hydrophilic fiber components.

**Figure 3:**
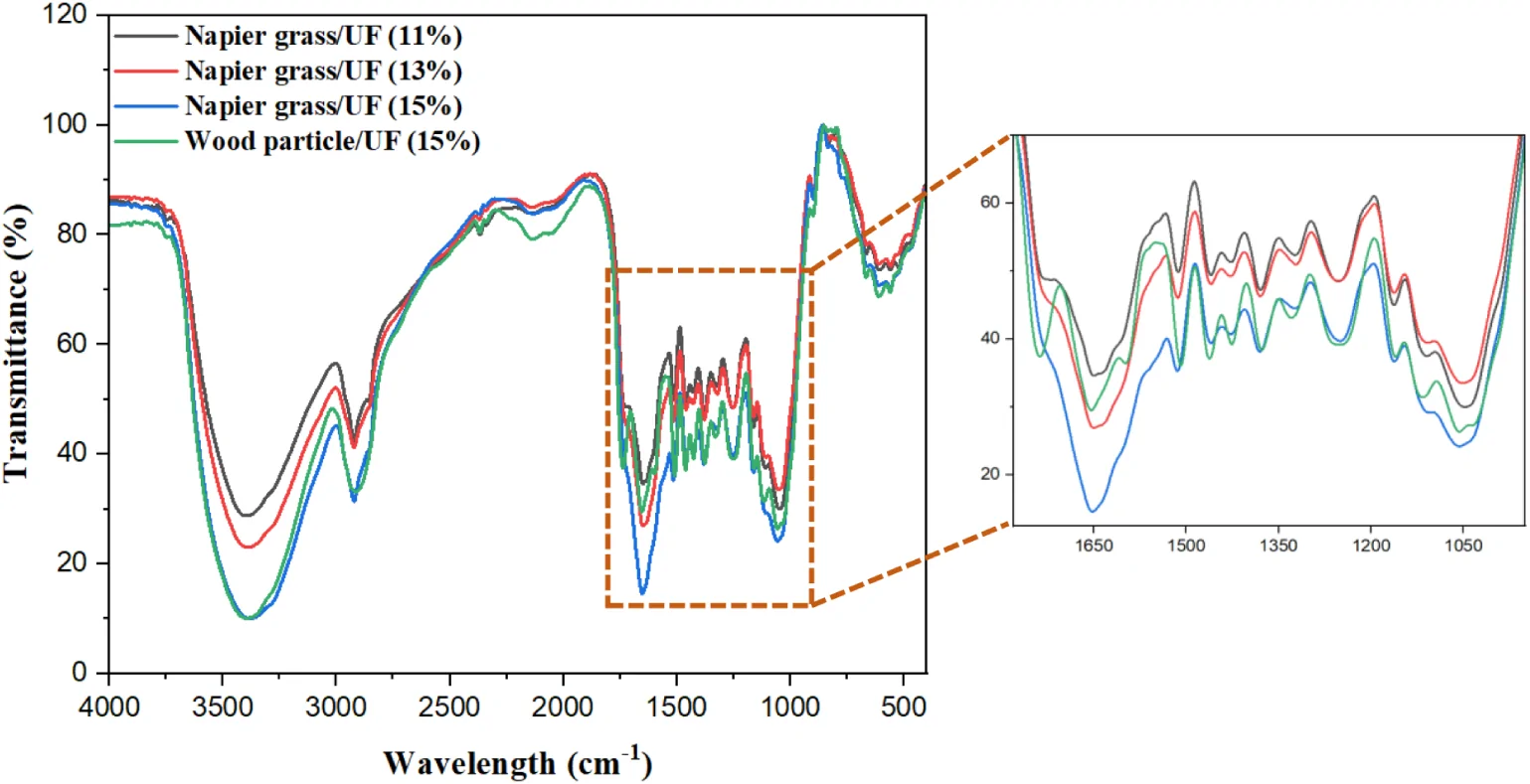
FTIR spectra of Napier grass particleboards with different UF resin contents (11%, 13%, and 15%) and wood particleboard (15% UF).

Although no new covalent functional groups or chemical bonds were detected between the fibers and UF resin, which is expected since UF bonding is primarily governed by physical interactions, including hydrogen bonding, secondary interactions, and mechanical interlocking. FTIR remains a valuable tool for evaluating the degree of interaction in particleboards. Variations in band intensity and relative band prominence provide evidence of resin distribution, curing efficiency, and the extent to which fiber surfaces are coated or embedded within the UF polymer matrix. These spectral trends correlate well with the physical and mechanical results, where increased resin content enhanced interfacial adhesion, reduced water absorption, and improved dimensional stability. This indicates that the performance of the composites is strongly governed by structure–property relationships arising from fiber chemistry and resin network formation. The FTIR findings confirm that the improvements observed in the mechanical and hygroscopic behavior of Napier grass particleboards arise from enhanced physical adhesion and resin coverage rather than chemical modification of the fibers. The comparison with wood particleboard provides a useful performance reference under identical processing conditions.

### 3.5 Mechanical Properties

The flexural modulus (MOE) of Napier grass particleboards manufactured with varying UF resin contents, alongside a wood particleboard fabricated at 15% resin content is presented in Figure 4a. The MOE of Napier-based boards increased markedly from 1002.66 MPa at 11% UF to 1629 MPa at 13% UF, indicating improved load transfer efficiency as the adhesive more effectively bonded adjacent particles. However, a decline in MOE was observed at 15% UF (1320 MPa), suggesting that excessive resin content did not further enhance stiffness. This nonlinear behavior is commonly reported for lignocellulosic composites, where the adhesive initially improves stress transfer but, beyond an optimal threshold, may cause embrittlement, reduced fiber swelling compatibility, or microcrack initiation within overpenetrated cell walls.^49^ High-viscosity UF can infiltrate lumens and thin cell walls during hot pressing. Excessive penetration stiffens localized regions and reduces the natural compliance of the fibrous structure, thereby diminishing the board’s ability to elastically resist bending. Additionally, elevated resin loadings can interfere with the intrinsic molecular ordering of lignocellulosic components; Yang et al. (2024) reported that excessive UF deposition reduces the crystallinity index of plant fibers, ultimately decreasing stiffness and the elastic response.^50^ This aligns with the current finding where 15% UF resulted in reduced MOE despite higher resin content. Comparatively, the wood particleboard bonded with 15% UF exhibited a much higher MOE (2864 MPa), reflecting superior stiffness relative to Napier-based boards. This difference is attributed to the higher density, lower porosity, and more uniform geometry of wood particles, which promote better compaction and more efficient stress transfer during bending. As demonstrated, engineered wood particles form stronger interparticle contact zones and develop a more homogeneous stress distribution under flexural loading, resulting in higher MOE values.^51^ The lower stiffness of Napier grass boards can also be associated with the inherent presence of parenchyma and vascular bundles, which reduce structural rigidity compared to wood. Previous work has shown that surface modification of Napier grass such as alkali treatment removes extractives and hemicellulose while exposing additional cellulose fibrils, thereby enhancing interfacial adhesion and improving mechanical properties.^52^ This suggests that pretreatments may narrow the performance gap between Napier grass and wood-based particleboards in future studies. Additionally, one-way ANOVA revealed a significant effect of formulation on MOE (*p* < 0.001). Tukey’s HSD test showed that the wood particleboard exhibited significantly higher MOE than all Napier grass particleboards, while the 13% UF formulation showed significantly higher MOE than the 11% UF board. No significant differences were observed between the 13% and 15% UF formulations or between the 15% and 11% UF formulations.

**Figure 4:**
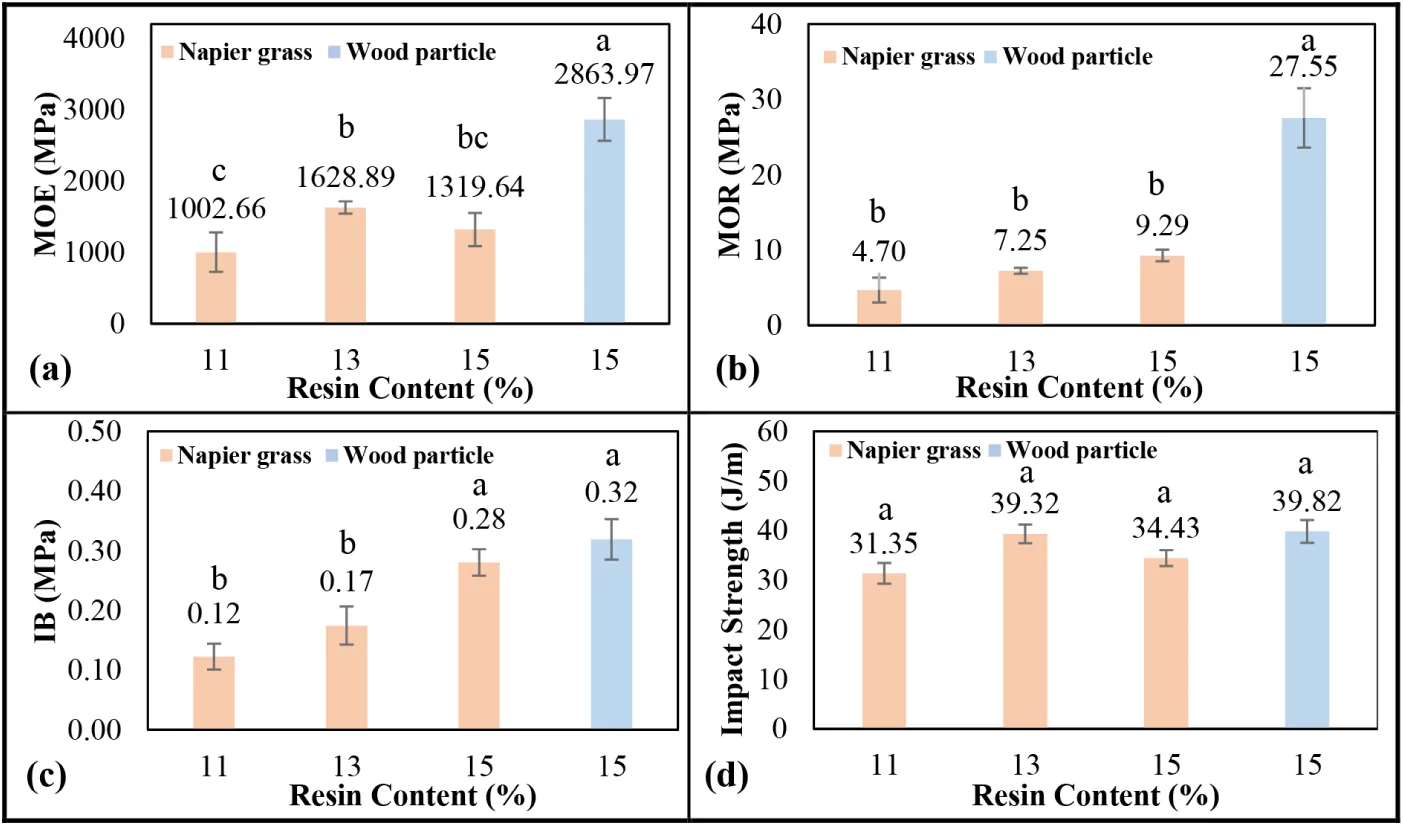
Mechanical performance of Napier grass particleboards with different UF resin contents (11%, 13%, and 15%) compared with wood particleboard at 15% UF (a) Modulus of elasticity (MOE), (b) Modulus of rupture (MOR), (c) Internal bonding strength (IB), and (d) Impact strength. Error bars represent standard deviation (*n* = 3). Different letters above the bars indicate significant differences according to Tukey’s HSD test (*p* < 0.05).

The modulus of rupture (MOR) of Napier grass particleboards produced with different UF resin contents, alongside a 15% UF wood particleboard for comparison (Figure 4b). The MOR of Napier-based boards increased substantially from 4.70 MPa at 11% UF to 9.29 MPa at 15% UF, indicating that resin content strongly influences the flexural strength of the composite. This trend is consistent with the well-established role of UF resin as a load-bearing matrix that bridges adjacent particles, enhances stress transfer, and reduces crack initiation under bending forces. At lower resin content, insufficient adhesive distribution leads to weak interparticle bonding and higher susceptibility to local deflection under bending, resulting in premature failure. Similar observations have been reported for lignocellulosic composites, where inadequate resin coverage is directly associated with reduced bending strength.^53^ The digital microscope images (Figure 5) corroborate this behavior. When compared to the wood particleboard, the Napier grass boards exhibited substantially lower MOR, with the wood board achieving 27.55 MPa at the same resin loading. The superior performance of wood particles can be attributed to their higher intrinsic density, more uniform geometry, lower parenchymal content, and better affinity with UF adhesives factors that promote stronger mechanical interlocking and more homogeneous stress distribution. The inherently more porous anatomical structure of Napier grass, combined with its higher hemicellulose content, contributes to weaker fiber–matrix compatibility and greater moisture-related instability, which further affects bending performance. Thus, although higher resin content improves MOR, the structural limitations of Napier grass remain a determining factor. One-way ANOVA revealed a significant effect of formulation on MOR (*p* < 0.001). Tukey’s HSD test showed that the wood particleboard exhibited significantly higher MOR than all Napier grass particleboards, whereas no significant differences were observed among the Napier grass particleboards prepared with different UF resin contents.

**Figure 5:**
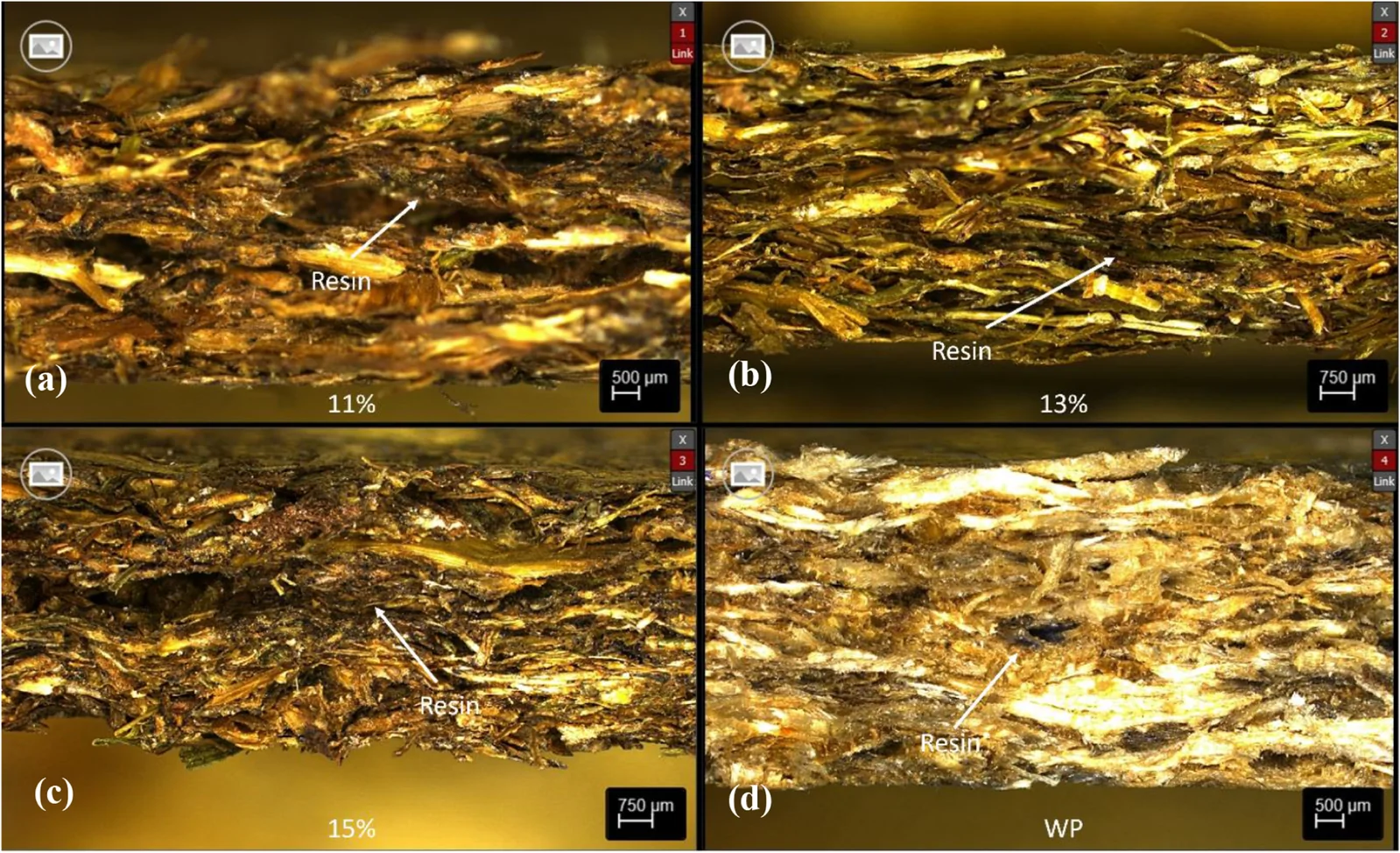
Digital microscopy images of fractured surfaces of Napier grass particleboards with different UF resin contents at: (a) 11%, (b) 13%, (c) 15%, and (d) wood particleboard at 15% UF.

Internal bonding (IB) strength of Napier grass particleboards fabricated with varying UF resin contents, alongside a 15% UF wood particleboard for comparison (Figure 4c). The IB of Napier grass boards increased markedly from 0.12 MPa at 11% UF to 0.28 MPa at 15% UF, indicating that resin content plays a critical role in enhancing interparticle cohesion. This improvement is attributed to the greater availability of adhesive to wet particle surfaces, fill voids, and establish continuous bonding bridges within the board structure. As adhesive coverage becomes more uniform and penetration into the porous biomass increases, the number of effective interfacial bonding sites rises, resulting in higher tensile resistance perpendicular to the panel surface. Similar adhesive–interface behavior has been widely reported in UF-bonded lignocellulosic composites.^18,38^ The progressive increase in IB also reflects enhanced resin distribution efficiency at higher UF loadings. Improved wetting and flow characteristics enable deeper adhesive penetration into lumens and cell wall cavities, strengthening the mechanical interlocking between particles. This effect is consistent with previous studies showing that adequate resin penetration is a dominant factor governing perpendicular tensile strength in biomass-based particleboards.^3^ Despite this improvement, the IB value of the Napier grass board at 15% UF (0.28 MPa) remained lower than that of the wood particleboard (0.32 MPa). The reduced bonding efficiency of Napier grass can be attributed to several intrinsic factors. First, its higher hemicellulose content and more heterogeneous parenchyma-rich structure led to uneven adhesive uptake and poorer surface wetting. Second, the higher amorphous cellulose fraction and the presence of silicaceous tissues reduce chemical compatibility with UF resin, limiting the formation of strong adhesive joints. Similar reductions in bond quality have been observed in nonwood biomass composites, especially those containing high proportions of vascular bundles or extractives that interfere with UF curing reactions.^54^ Overall, while increasing UF content improved the IB performance of Napier grass particleboards, the material’s anatomical and chemical characteristics inherently restrict optimum fiber–matrix interaction compared with conventional wood particles. Targeted fiber modifications such as alkaline treatment, removal of waxy layers, or coupling-agent treatment may be required to further enhance IB strength and achieve performance comparable to wood-based panels. One-way ANOVA revealed a significant effect of formulation on internal bond strength (IB) (*p* < 0.001). Tukey’s HSD test indicated that the 15% UF Napier grass particleboard and the wood particleboard were not significantly different from each other, whereas both exhibited significantly higher IB values than the 11% and 13% UF formulations.

Impact strength of Napier grass particleboards produced with different UF resin contents, alongside a 15% UF wood particleboard for comparison (Figure 4d). The impact strength of Napier grass boards increased from 31.35 J/m at 11% UF to a maximum of 39.32 J/m at 13% UF, suggesting that moderate increases in resin content enhance the board’s ability to dissipate energy under rapid loading. This improvement corresponds to better interfacial adhesion between particles and UF resin, which reduces microcrack formation and allows the composite to absorb more energy before catastrophic failure. A similar trend has been reported in lignocellulosic particleboards where improved fiber–matrix adhesion directly correlates with higher impact resistance.^55^ However, a slight decline in impact strength was observed at 15% UF (34.43 J/m). Although the reduction is modest, it indicates that excessive resin can introduce localized brittleness due to oversaturation of adhesive in certain regions, leading to stress concentration and reduced crack-arresting capability. This behavior is consistent with the tendency of UF to become more brittle at higher solid contents, which limits its ability to bridge cracks effectively during high-speed deformation.^56^ The slight drop also suggests that beyond an optimal resin level, the stiffened matrix may reduce energy absorption capacity despite improved bonding.

Although the 13% UF board exhibited the highest impact strength, the 15% formulation was selected as the overall optimum due to its superior performance in other critical properties including dimensional stability, IB strength, and MOR. When comparing the optimum 15% UF Napier grass board to the 15% UF wood particleboard, the wood panel exhibited substantially higher impact strength (39.82 J/m). This difference may be related to variations in particle morphology, internal structure, and energy dissipation behavior between the two lignocellulosic materials.^33^ In contrast, the more porous and heterogeneous microstructure of Napier grass results in lower intrinsic toughness and contributes to reduced impact performance despite adequate resin bonding. One-way ANOVA indicated that formulation had no significant effect on impact strength (p = 0.354). Accordingly, no statistically significant differences were observed among the Napier grass particleboards and the wood particleboard according to Tukey’s HSD test (*p* > 0.05). Overall, the results demonstrate that while Napier grass particleboards show promising impact resistance, their impact performance remains slightly lower than that of wood particleboards under identical processing conditions. Fiber modification, precompression, or blending with denser particles may further enhance their impact performance for structural applications.

### 3.6 Digital Microscopy

The digital microscopy images of Napier grass and wood particleboards (Figure 5), highlighting the fracture morphology and adhesive distribution at different UF resin contents. Clear structural differences are observed between the two raw materials as well as among the Napier boards prepared with varying resin levels. At 15% UF resin, the Napier grass particleboard displays numerous particle pull-outs and visible gaps at the particle–particle interfaces, indicating weaker interlocking and limited cohesive strength. In contrast, the wood particleboard at the same resin loading shows a markedly more compact structure, with closely packed particles and minimal pull-outs, reflecting stronger mechanical interlocking and more effective stress transfer across the matrix–fiber interface. The micrographs also reveal how resin content influences the internal structure of Napier grass particleboards. Boards fabricated with 11% and 13% UF show distinct voids, microcracks, and regions where resin distribution is incomplete. These unbonded zones serve as preferential pathways for crack initiation during flexural loading, thereby lowering MOR. Conversely, the 15% UF board demonstrates a noticeably denser and more consolidated microstructure, with improved resin coverage and fewer interparticle voids. This enhanced packing is consistent with the corresponding increase in mechanical performance, confirming that adequate adhesive penetration is essential for forming a continuous load-bearing network. Such morphology–property correlations are well established in particleboard literature: a uniform internal structure with minimal defects effectively delays crack propagation, reduces stress concentration, and leads to higher bending strength and improved global integrity.^57,58^ The microscopic observations substantiate the mechanical results and highlight the role of resin distribution and particle compactness in achieving reliable performance in Napier grass particleboards.

### 3.7 Thermal Properties

The thermogravimetric behavior of Napier grass particleboards manufactured with 11%, 13%, and 15% UF resin contents, alongside a 15% UF wood particleboard is presented in Figure 6 (a and b). All samples exhibited the characteristic thermal degradation profile of lignocellulosic composites, comprising an initial low-temperature mass loss followed by major devolatilization and char-forming stages. The small weight loss observed below 200 °C corresponds to moisture evaporation and the release of low-molecular-weight volatiles. This behavior is typical in biomass-based boards due to the hydrophilic nature of cellulose and hemicellulose.^50^ The onset of major decomposition for Napier grass boards occurred between 270–280 °C, increasing slightly with resin content (270.10 °C for 11% UF, 279.39 °C for 13% UF, and 278.36 °C for 15% UF). This upward shift in initial degradation temperature indicates improved thermal stability with higher adhesive content, as increased resin loading promotes the formation of a cross-linked UF network that restricts polymer chain mobility and delays thermal decomposition. The enhanced thermal resistance can be attributed to the thermosetting nature of UF resin, where curing results in a three-dimensional network structure that increases resistance to thermal scission and volatilization. Enhanced thermal resistance with increased UF content has also been reported in other UF-bonded agricultural residue composites due to improved structural consolidation and limited oxygen diffusion into the matrix.^59^

**Figure 6:**
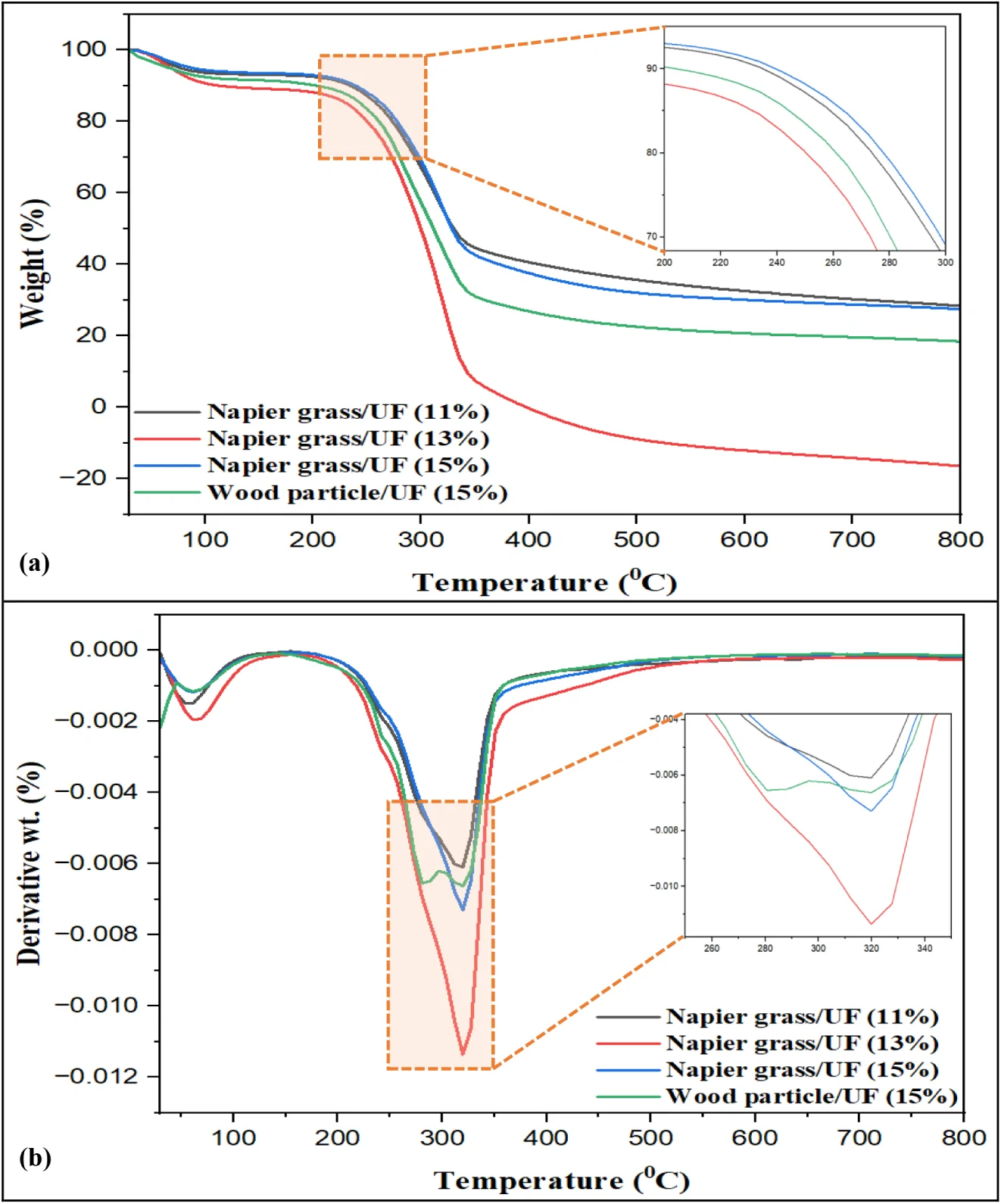
(a) Thermogravimetric analysis (TGA), and (b) derivative thermogravimetry (DTG) curves of Napier grass particleboards fabricated with 11%, 13%, and 15% UF resin content, compared with wood particleboard at 15% UF.

Compared with Napier grass boards, the wood particleboard displayed a slightly lower onset temperature (261 °C), suggesting earlier thermal degradation. This difference may be attributed to the higher hemicellulose fraction and more reactive amorphous regions in wood, which initiate decomposition sooner. In contrast, Napier grass contains higher silica, extractives, and mineral inclusions, which have been shown to enhance thermal resistance and delay pyrolysis in nonwood biomass.^20^ A notable distinction was observed in the residual char content. The Napier grass board with 15% UF exhibited the highest char residue (27.75%), significantly exceeding that of the wood particleboard (18.74%). This suggests a greater proportion of thermally stable inorganic ash and fixed carbon in Napier grass, consistent with previous findings reporting ash contents of 8–12% in Napier grass compared with only 1–3% for most hardwood species.^60^ The higher char yield may also be associated with lignin-rich fractions and inorganic silica, which promote carbonaceous residue formation and act as thermal barriers during decomposition. Higher char residue is advantageous for fire retardancy and thermal shielding, making Napier grass-based composites potentially favorable for applications requiring improved thermal resistance. The TGA results confirm that Napier grass particleboards exhibit thermal stability comparable to or higher than wood-based boards, particularly at higher resin contents. This reflects the combined effects of UF network formation, lignocellulosic composition, and mineral content, establishing a clear structure–thermal property relationship in the composite system

## 4 Conclusion

This study demonstrates the technical feasibility of Napier grass as a sustainable lignocellulosic biomass feedstock for physicochemical conversion into particleboard materials. By varying urea–formaldehyde (UF) resin content from 11–15 wt %, particleboards with improved mechanical performance, interfacial bonding, and dimensional stability were obtained. The formulation containing 15% UF provided the most balanced properties, yielding the highest modulus of rupture and internal bond strength, along with reduced water absorption, thickness swelling, and enhanced thermal stability. Statistical analysis further confirmed that resin content significantly influenced MOE, MOR, and internal bond strength. Although the mechanical properties of Napier grass particleboards remained slightly lower than those of conventional wood-based boards, the comparable performance trends and acceptable property range confirm its viability as a nonwood raw material for interior, nonstructural applications. These improvements are attributed to enhanced interfacial interactions and resin distribution within the particleboard structure, which improved stress transfer efficiency, moisture resistance, and thermal behavior, establishing a clear structure–property relationship in the composite system. The successful conversion of Napier grass into a functional composite product highlights its potential as an underutilized biomass resource capable of supporting the development of low-carbon, renewable, nonfood materials. While this study provides valuable insight into the structure–property relationships of Napier grass particleboards, it is limited to a single board density and UF adhesive system, and long-term durability and environmental performance were not investigated. Future research should evaluate formaldehyde emissions from Napier grass particleboards, as well as explore formaldehyde-free or biobased adhesive systems, fiber surface modification to further improve matrix compatibility, and complete life cycle assessment (LCA) to quantify environmental benefits relative to wood-derived boards. Overall, this work contributes to advancing biomass valorization strategies and provides insight into the role of lignocellulosic composition and resin network formation in governing composite performance, demonstrating a practical pathway for transforming agricultural biomass into value-added engineered materials within a sustainable materials framework, thereby supporting the diversification of sustainable raw materials for future wood-based composite manufacturing.

## Data Availability

Data will be made available upon request.
